# Advance Care Planning Initiatives in Critical Care Centers in Japan: A Case Report

**DOI:** 10.1002/ccr3.9703

**Published:** 2025-01-07

**Authors:** Yuni Kamimura, Michihiro Tsubaki, Jun Hattori, Marina Oi, Takaaki Maruhashi, Haruyo Ota, Miho Oki, Kota Takanashi, Maika Takahashi, Yoko Sakurai, Yasushi Asari

**Affiliations:** ^1^ Department of Nursing, Emergency and Disaster Medical Center Kitasato University Hospital Sagamihara Japan; ^2^ Department of Clinical Nursing Kitasato University School of Nursing Sagamihara Japan; ^3^ Department of Emergency and Critical Care Medicine Kitasato University School of Medicine Sagamihara Japan

**Keywords:** advance care planning, case report, critical care centers, electronic patient‐reported outcomes, emergency department

## Abstract

This report examines the promotion of advance care planning (ACP) for patients admitted to critical care centers and discharged to home. Emergency transport experience allows patients and their families to realistically discuss her ACP.

## Graphical abstract

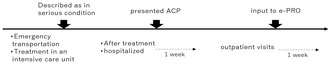

## 1 Introduction

In Japan, where the aging population is increasing, responding to emergency medical and end‐of‐life care for older adults is difficult. The number of out‐of‐hospital cardiac arrest cases involving older adults requiring ambulance transportation is increasing annually [1]. Even in cases where patients have indicated their intention of Do Not Attempt Resuscitation (DNAR) in advance at home medical care or elderly welfare facilities, families and facility staff who have witnessed sudden cardiac arrest may request an ambulance. The patient's advance directives, advance care plans, and discussions with the family are important for respecting patient preferences [2]. To this end, family acceptance of the patient's wishes and a mechanism to share information with the family and medical personnel are necessary. Advance care planning (ACP) is attracting attention, but despite its potential benefits, It is also clear from previous studies that ACP is not widely used among the elderly [3]. According to a questionnaire survey [4] on ACP released by the Ministry of Health, Labor, and Welfare in 2018, the idea of ACP is not very popular, and the same is true in the Japanese society. The most common reason for this is “There was no opportunity for discussion” (56%) [4]. Dissemination of the concept of ACP and provision of opportunities for discussion at appropriate times are necessary to provide patient‐centered medical care. It should be noted that ACP is subject to change according to the situation and patients' state of mind. In other words, continuity and flexibility are required, as is a mechanism for sharing changes in real time by medical professionals. However, few medical institutions recognize or present content that should be discussed in the ACP. Therefore, we introduced an electronic patient‐reported outcome application (e‐PRO) to continuously implement ACP after discharge and share changes in intentions in real time. In this application, patients enter electronic information by answering 10 questions about their expectations and satisfaction with symptom management and treatment and share it with their families and medical professionals via a cloud system. The introduction of this application guides the content to be discussed at the ACP and provides an opportunity to share patient's intentions with their families. This report presents the significance of promoting ACP in the emergency department based on a case of medical personnel providing an opportunity to think about ACP to a patient who experienced ambulance transportation and was discharged.

## 2 Case History

A female patient in her 77 years old was admitted with septic shock associated with urinary tract infection caused by left urinary tract stones. She lives with her eldest daughter, who is a caregiver, and her grandson, who is in high school. Her eldest daughter was a key person in her treatment.

## 3 Investigations and Treatment

The doctor in charge of treatment explained the patient's condition and treatment method. The general explanation in such cases is that this condition has a mortality rate of approximately 30%. The doctor presented her treatment options, and her eldest daughter signed a consent form saying, “I will leave it all to the doctor.” Her eldest daughter decided on her behalf. The postoperative course was favorable, and the patient was discharged after approximately 1 week of hospitalization. After the surgery, the attending physician explained the patient's condition, presented the patient with ACP, and explained the procedure. After receiving an explanation from the attending physician, the patient responded, “I have never thought about it seriously, so it is a very good opportunity.” “It is a miracle that she recovered enough to talk this time,” her eldest daughter said, acknowledging the patient's condition. The nurses in the inpatient ward indicated the patient's intention to go home and began strenuous rehabilitation.

After discharge from the hospital, antibiotic therapy was initiated as an outpatient treatment. The eldest daughter was always present during outpatient visits, and the patient used a wheelchair to visit the hospital. The emergency outpatient nurse asked the patient, “Have you ever thought about how you would approach the end of your life and how you would the rest of your life, and have you talked to your family about it? The patient replied,” My husband died suddenly the day after he was told he was fine during a consultation, and I regretted it. “The eldest daughter said,” After my father's sudden death, I started talking to my mother about what to do in case something happened. We discussed organ donation if there is something that can be used. I haven't really talked about anything else. “The nurse was informed that the patient had been discussing her willingness to donate organs to other patients in need.”

## 4 Outcome and Follow‐Up

The emergency department nurse confirmed that the patient had consented to participate in ACP. At this point, input via e‐PRO was requested. The e‐PRO consists of 10 questions (Table 1).

**TABLE 1:** Questions about ACP.

[What beliefs do you hold dear in life?] [Which of the following situations would make you hopeless in life?] [What are your thoughts on having an incurable disease?] [What kind of treatment would you like to receive?] [Have you received detailed explanations about your health and illness and are you able to understand it?] [You will not be able to make decisions for yourself due to dementia or brain disorders. What are your hopes when the time comes?] [Where would you like to recuperate when the time comes?] [Do you have anyone you can entrust your thoughts to?] [Did you have any opportunities to discuss your feelings with your family during your hospitalization?] [What did the medical staff ask you during your hospitalization?]

**FIGURE 1:**
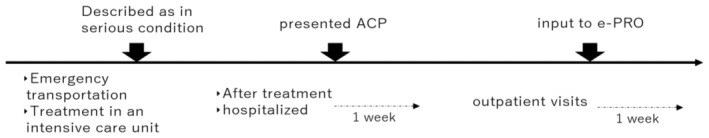
Intervention process.

The data entered can be shared with family members within the app and with medical personnel. The patient and her eldest daughter had the opportunity to share, for the first time, the specific content that should be discussed for ACP, in addition to their intention to donate organs. The intervention process for the ACP is illustrated in Figure 1.

## 5 Discussion

### 5.1 ACP Promotion in the Emergency Department

#### 5.1.1 The Presentation of the Content to Be Discussed at the ACP

The patient was transported in a state of disturbed consciousness, and the treatment was advanced by the eldest daughter's surrogate intention. Although this is part of the process of providing tertiary emergency medical care, it can be assumed that the family will experience a heavy emotional burden if the patient is forced to make a life‐or‐death decision. Throughout history, most treatment policies in Japan have been decided by families. There are also reports that decisions are made mainly by the eldest son, with no consideration given to the wishes of other family members [5]. When the eldest daughter came to the hospital, she said, “I will leave everything to the medical staff.” From this, we predicted that it would take time for her eldest daughter to realize her role as an advocate of the patient's feelings because she was upset. Alternatively, she may not have had enough time, and made a surrogate decision considering the patient's best interests [6]. The only discussion in the family so far was about organ donation, and there was no discussion about the intention to receive treatment when she became ill or how to spend her time in the future. In other words, in situations where there is no clear visualization of the process that the patient or their family will follow, the content of the discussion will be limited, and there will be no concreteness or effectiveness. The patient was then discharged. However, immediately after discharge from the hospital, she was wheelchair‐bound and thus experiencing a situation in which she could not live without assistance. Responses to questions about ACP after discharge differed from those on admission. Thus, because ACP changes according to the patient's situation, it is necessary to assume various situations. Therefore, the support of medical staff is needed [7] for presenting specific content to facilitate discussions with the family. In addition, we believe that even if a patient is unable to express their intentions, the family is more likely to be able to choose treatment based on the patient's intentions [8].

#### 5.1.2 Timing of Introduction of ACP in the Emergency Department

The timing of ACP intervention is also important. As in this case, having a nurse conduct an interview when the patient became an outpatient after overcoming a crisis provided an opportunity for the patient's family to think realistically about ACP [9, 10]. Sharing ACP details among patients, their families, and medical staff in the emergency medical field is important. Promoting ACP may help reduce the psychological burden on patients' families who make surrogate decisions [11, 12], lead to the provision of patient‐oriented medical care [7] and protect human dignity.

### 5.2 Introduction of the Electronic Patient‐Reported Outcome Type Application

In clinical practice, patient‐reported outcomes are the information provided directly by the patient and have been reported to help improve patient diagnosis and treatment. However, there have been no reports on the introduction of e‐PROs into ACP. The patient in this report was an older adult. Relatively few older adults are good at using electronic devices, and it can be predicted that interventions by others are necessary. Therefore, it is important to discuss ACP with their family members. Therefore, introducing ePROs into ACP inevitably requires family intervention. In Japan, even if the patient had expressed their intentions, the family may be unaware of this, making it clear that ACP was not carried out [5]. We believe that the introduction of e‐PRO will be a good opportunity for discussions between patients and their families. Furthermore, it is important for nurses to instruct patients to share the information they have entered with their families, which is one of their roles [13].

## 6 Conclusion

Considering this case, two points are of note regarding the significance of introducing ACP in the emergency department.

It has been suggested that promoting ACP in the wake of emergency transportation, which affects the prognosis of life, will enable patients and their families to discuss this concretely and realistically. However, introducing ACP requires patients and their families to understand its benefits and intentions fully. A system in which nurses proactively intervene should be considered in the future.

Furthermore, ACP should always include consideration of the timing and flexibility of the intervention. The e‐PRO‐type application used in this initiative allows for regular input. We believe that the patients' certainty of the provided information can be increased by performing ACP according to the patient's situations.

## Author Contributions

**Yuni Kamimura:** conceptualization, data curation, formal analysis, investigation, methodology, project administration, validation, visualization, writing – original draft, writing – review and editing. **Michihiro Tsubaki:** methodology, writing – review and editing. **Jun Hattori:** conceptualization, project administration. **Marina Oi:** data curation. **Takaaki Maruhashi:** funding acquisition, supervision, writing – original draft. **Haruyo Ota:** data curation. **Miho Oki:** data curation. **Kota Takanashi:** data curation. **Maika Takahashi:** data curation. **Yoko Sakurai:** data curation. **Yasushi Asari:** writing – review and editing.

## Ethics Statement

The Ethics Committee of the Kitasato University Hospital approved this study. (Approval number: B21‐187).

## Consent

Written informed consent for the publication of patient data was obtained from the patient.

## Conflicts of Interest

The authors declare no conflicts of interest.

## Permission to Reproduce Material From Other Sources

This is an open access article under the terms of the Creative Commons Attribution License, which permits use, distribution, and reproduction in many mediums, provided the original work is properly cited.

## Data Availability

The authors have nothing to report.
